# Graphene oxide enhances alginate encapsulated cells viability and functionality while not affecting the foreign body response

**DOI:** 10.1080/10717544.2018.1474966

**Published:** 2018-05-19

**Authors:** Jesús Ciriza, Laura Saenz del Burgo, Haritz Gurruchaga, Francesc E. Borras, Marcella Franquesa, Gorka Orive, Rosa Maria Hernández, José Luis Pedraz

**Affiliations:** a Biomedical Research Networking Center in Bioengineering, Biomaterials and Nanomedicine, CIBER-BBN, Vitoria-Gasteiz, Spain;; b NanoBioCel Group, Laboratory of Pharmacy and Pharmaceutical Technology, Faculty of Pharmacy, University of the Basque Country, UPV/EHU, Vitoria-Gasteiz, Spain;; c REMAR-IVECAT Group, Health Science Research Institute Germans Trias i Pujol, Badalona, Spain;; d Department of Cell Biology, Physiology and Immunology, Universitat Autònoma de Barcelona, Bellaterra, Spain;; e Nephrology Service, Germans Trias i Pujol University Hospital, Badalona, Spain

**Keywords:** Graphene oxide, cell microencapsulation, stem cells, erythropoietin, immune response

## Abstract

The combination of protein-coated graphene oxide (GO) and microencapsulation technology has moved a step forward in the challenge of improving long-term alginate encapsulated cell survival and sustainable therapeutic protein release, bringing closer its translation from bench to the clinic. Although this new approach in cell microencapsulation represents a great promise for long-term drug delivery, previous studies have been performed only with encapsulated murine C_2_C_12_ myoblasts genetically engineered to secrete murine erythropoietin (C_2_C_12_-EPO) within 160 µm diameter hybrid alginate protein-coated GO microcapsules implanted into syngeneic mice. Here, we show that encapsulated C_2_C_12_-EPO myoblasts survive longer and release more therapeutic protein by doubling the micron diameter of hybrid alginate-protein-coated GO microcapsules to 380 µm range. Encapsulated mesenchymal stem cells (MSC) genetically modified to secrete erythropoietin (D1-MSCs-EPO) within 380 µm-diameter hybrid alginate-protein-coated GO microcapsules confirmed this improvement in survival and sustained protein release *in vitro*. This improved behavior is reflected in the hematocrit increase of allogeneic mice implanted with both encapsulated cell types within 380 µm diameter hybrid alginate-protein-coated GO microcapsules, showing lower immune response with encapsulated MSCs. These results provide a new relevant step for the future clinical application of protein-coated GO on cell microencapsulation.

## Introduction

Alginate microencapsulated cell-based therapies can provide the sustained release of therapeutic products in the treatment of a variety of diseases. Cells are considered microencapsulated when they are entrapped within a semipermeable polymer matrix at the micrometer scale, preventing both migration of the entrapped cells and invasion of host immune responders. The pores of the encapsulating polymer are large enough to permit the ingress of nutrients, oxygen, and electrolytes and the egress of molecules produced by entrapped cells, such as hormones, metabolites, and waste products. Larger host immune responders such as cells, immunoglobulins and complement cannot penetrate the microcapsules walls and are, therefore, hindered from interacting with surface antigens on microencapsulated cells (Orive et al., 2005; Prakash & Jones, 2005), allowing their immunoisolation.

Currently, several challenges in cell therapy using microencapsulated cells need to be overcome, such as the decrease of the dying cell number inside the microcapsules or the enhancement of the sustained protein release from encapsulated cells, achieving long-lasting treatments. In this regard, we combined cell microencapsulation technology with graphene oxide (GO), a highly oxidized form of chemically modified graphene, consisting of a single atom thick layer of graphene sheets with carboxylic acid, epoxide, and hydroxyl groups (Goenka et al., 2013). We incorporated different concentrations of GO into 160 µm diameter alginate microcapsules, showing that GO concentrations between 25 and 50 µg/ml increased the viability, metabolic activity, membrane integrity, and erythropoietin (EPO) release of encapsulated murine C_2_C_12_ myoblasts genetically engineered to secrete murine erythropoietin (C_2_C_12_-EPO) (Ciriza et al., 2015), improving even more by the formation of a protein bio-corona with fetal bovine serum (FBS) (Saenz Del Burgo et al., 2017). This increment in EPO release was reflected *in vivo* by an enhancement of hematocrit levels after implantation in syngeneic mice of 160 µm diameter hybrid alginate-protein-coated GO (50 µg/ml) microcapsules containing C_2_C_12_-EPO myoblasts (Saenz Del Burgo et al., 2017). However, other cell types should be assessed both *in vitro* and *in vivo* (Ciriza et al., 2015), to confirm the successful results demonstrated by combining alginate microcapsule technology with GO.

Another challenge in cell therapy using microencapsulated cells is the size of microcapsules. The combination of alginate microencapsulation and GO initially was performed within 160 µm diameter microcapsules (Ciriza et al., 2015; Saenz Del Burgo et al., 2017) because small-sized microcapsules showed better surface/volume ratio, reduced mass transport limitations, and enhanced biocompatibility (Robitaille et al., 1999; Sugiura et al., 2007), with faster ingress and egress of molecules (Wilson & Chaikof, 2008; Sakai & Kawakami, 2010). Although diameters from 100 µm of alginate microcapsules have been widely used for *in vivo* applications, such as controlled drug release or systems for tissue regeneration (Whelehan & Marison, 2011; Lee & Mooney, 2012), bigger diameters between 300 µm and 1 mm have been more extensively evaluated in clinical application for the last four decades, such as the immune isolation of donor pancreatic islets for the treatment of type-1 diabetes (Lim & Sun, 1980). In this sense, it is relevant to determine the behavior of encapsulated cells within hybrid alginate-protein-coated GO microcapsules with diameter bigger than 300 µm.

Finally, the foreign body response against biomaterial is an important challenge to overcome. The immune rejection of alginate encapsulated cells is not always completely bypassed by alginate microcapsules. For example, CD4^+^ T cells, B cells, and macrophages can secrete immune molecules and complement that traverse microcapsules destroying the inner encapsulated xenograft cells (Kobayashi et al., 2006). Moreover, the biomaterial is often immune recognized, initiating a cascade of cellular processes to lead the foreign body reaction (Anderson et al., 2008; Williams, 2008). These processes consist on inflammation, formation of fused macrophages that generate foreign body giant cells, and fibrosis, that finally builds up a 100-µm thick fibrotic tissue enveloping the implanted biomaterial and affecting the functionality of the device (Ratner, 2002). In this regard, mesenchymal stem cells (MSCs) have arisen great interest in the last decades, due to their immunomodulatory properties (Rasmusson, 2006; Uccelli et al., 2006). They have been examined in a variety of animal models related to alloreactive immunity (organ and stem cell transplantation), autoimmunity, or tumor immunity. The first systemic infusion of allogeneic baboon-bone marrow-MSCs prolonged allogeneic skin grafts survival from 7 to 11 d, compared to animals non-infused with MSCs (Bartholomew et al., 2002). Interestingly, MSC immunomodulatory capacity is altered in 3-D culture systems, together with phenotypic cellular changes, having high potential for tissue engineering and cellular therapies. For example, MSCs within alginate hydrogels inhibit phytohemaglutinin-stimulated peripheral blood mononuclear cell proliferation more than monolayer-MSCs (Follin et al., 2015), or co-cultures of rat organotypic hippocampal slides with MSCs embedded into an alginate hydrogel, reduce TNF-α inflammation more than co-cultures with non-embedded MSCs (Stucky et al., 2015). MSCs, therefore, do not only directly participate in tissue repair and regeneration but also may modulate the host foreign body response toward the engineered construct, holding a great promise in tissue engineering.

In summary, three main challenges with hybrid alginate-protein-coated GO microcapsules remain untested: (1) the encapsulation with new cell types, (2) the effect of the microcapsule size, and (3) the circumvention of the foreign body reaction. Therefore, we aimed to study how increasing the diameter size of hybrid alginate-protein-coated GO microcapsules from 160 to 380 µm would affect the viability and functionality of encapsulated C_2_C_12_-EPO myoblasts, further studying this effect with encapsulated MSCs. Next, we compared the *in vivo* beneficial effects after implantation of encapsulated C_2_C_12_-EPO and MSCs genetically modified to secrete EPO (D1-MSCs-EPO) within both diameter size alginate-protein-coated GO alginate microcapsules into allogeneic mice, confirming a lack of foreign body reaction increment by the presence of GO, the microcapsules size or the encapsulated cell type.

## Material and methods

### Materials and reagents

GO 3 wt % was kindly provided by Graphenea Company (San Sebastian, Spain). The product was suspended in FBS (Gibco, Waltham, MA, USA) and sonicated for 1 h in order to obtain a higher percentage of monolayer flakes. Ultra pure low-viscosity (20–200 mPa*s) and high guluronic (LVG) sodium alginate (G/M ratio ≥1.5) with MW of 75–200 kDa was purchased from FMC Biopolymer (NovaMatrix, Sandvika, Norway). Poly-l-lysine hydrobromide (PLL, 15-30 kDa) was purchased from Sigma-Aldrich (St Louis, MO, USA).

The following monoclonal antibodies were purchased from BioLegend (San Diego, CA, USA) and used in flow cytometry: PE-Cy7 conjugated anti CD45 (I3/2.3), FITC-conjugated anti-CD3 (17A2), PE conjugated anti-CD4 (RM4-4), APC conjugated anti-CD8 (53-6.7), APC conjugated anti-CD11b (M1/70), PE conjugated anti-CD19 (6D5), APC-Cy7 conjugated anti-NK1.1 (PK136), PE-Cy7 conjugated Rat IgG2b, κ Isotype Ctrl (RTK4530), APC-Cy7 conjugated Mouse IgG2a, κ Isotype Ctrl (MOPC-173), FITC conjugated Rat IgG2b, κ Isotype Ctrl(RTK4530), PE conjugated Rat IgG2b, κ Isotype Ctrl (RTK4530), PE conjugated Rat IgG2a, κ Isotype Ctrl (RTK2758), APC conjugated Rat IgG2a, κ Isotype Ctrl (RTK2758), APC conjugated Rat IgG2b, κ Isotype Ctrl (RTK4530). Each antibody was carefully titrated and used at a concentration that gave the highest signal with the lowest background, following staining of splenocytes single cell suspensions.

### Cell culture

Murine C_2_C_12_ genetically engineered to secrete murine erythropoietin (C_2_C_12_-EPO) (Murua et al., 2009) were grown in T-flasks with Dulbecco’s modified Eagles’s medium (DMEM; Gibco) supplemented with inactivated 10% FBS (Gibco), 2 mM l-glutamine (Gibco) and 1% antibiotic/antimycotic solution (Gibco) at 37 °C in humidified 5% CO_2_/95% air atmosphere. Murine D1 MSCs engineered to secrete erythropoietin (D1-MSCs-EPO) (Gurruchaga et al., 2015) were grown with DMEM (Gibco) supplemented with 10% FBS (Gibco) and 1% penicillin/streptomycin solution (Gibco) at 37 °C in humidified 5% CO_2_ atmosphere. Cells were passaged every 2–3 d.

### Cell encapsulation

Sterile 1.5% and 1.87% alginate solutions were prepared by dissolving LVG alginate in 1% mannitol solution and filtered through a 0.20-µM syringe filter (Millipore, Billerica, MA, USA). GO was suspended in FBS (Gibco) at a concentration of 250 µg/ml and sonicated for 1 h. GO suspensions were mixed with 1.87% alginate solutions to a final concentration of 1.5% alginate and the following GO final concentrations: 50 µg/ml, 25 µg/ml, and 10 µg/ml. Murine C_2_C_12_-EPO cells and D1-MSCs-EPO cells were harvested using 0.25% trypsin-EDTA (Life Technologies, Carlsbad, CA, USA) and re-suspended into the sterile 1.5% alginate solutions containing 50 µg/ml, 25 µg/ml, or 10 µg/ml GO. As controls, C_2_C_12_-EPO cells and D1-MSCs-EPO cells were resuspended in a 1.5% alginate solution without GO. Cells were encapsulated at a density of 5 × 10^6^ cells/ml of alginate. Suspensions were extruded using a 5 ml sterile syringe through a disposable nebulizer in a pneumatic atomization generator (Bioencapsulation portable platform Cellena^®^) or an electrostatic atomization generator (Nisco^®^). The resulting alginate beads were maintained in agitation for 10 min in a CaCl_2_ solution (55 mM) for complete ionic gelation. Afterwards, they were ionically linked with 0.05% (w/v) PLL for 5 min and coated with 0.1% LVG alginate for another 5 min. Microcapsules were prepared at room temperature, under aseptic conditions and were cultured in C_2_C_12_-EPO and D1-MSCs-EPO media. The diameters (160 µm and 380 µm) and overall morphology of microcapsules were characterized using an inverted optical microscopy (Nikon TSM).

### Early apoptosis quantification

Early apoptosis of microencapsulated C_2_C_12_-EPO and D1-MSCs-EPO cells with alginate and 50 µg/ml, 25 µg/ml, and 10 µg/ml concentrations of GO was quantified with the Annexin-V-FITC Apoptosis Detection Kit (Sigma-Aldrich) at days 1 and 7 post-encapsulation. Microencapsulated cells in alginate matrices without GO were studied as controls. Briefly, 200 µl of microcapsules (10^6^ cells) were incubated with alginate lyase (Sigma-Aldrich) for 30 min at 37 °C to release the cells. The lysate was rinsed twice with DPBS and re-suspended in binding buffer consisting of 10 mM HEPES/NaOH, pH 7.5, containing 0.14 M NaCl and 2.5 mM CaCl2 (binding buffer) and stained with annexin V-FITC and propidium iodide for 10 min at room temperature protected from light. Unstained samples or stained only with annexin V-FITC or propidium iodide were used to establish the appropriate acquisition parameters for the analyzed samples. Fluorescence was determined in a BD FACS Calibur flow cytometer. Three independent samples were analyzed for each condition.

### Cell viability

Cell viability of microencapsulated cells in alginate matrices with 50 µg/ml, 25 µg/ml, and 10 µg/ml concentrations of GO was quantified with the LIVE/DEAD^®^ Viability/Cytotoxicity Kit (Invitrogen™) at days 1 and 7 post-encapsulation. Encapsulated cells in alginate without GO were studied as controls. Cells were released from microcapsules by incubation with alginate lyase for 30 min at 37 °C, and stained with 100 nM calcein AM and 8 µM ethidium homodimer-1 solution for 20 min at room temperature, protected from light. Unstained samples or stained only with 100 nM calcein AM or 8 µM ethidium homodimer-1 were ran as controls to establish the appropriate acquisition parameters for the analyzed samples. Fluorescence was quantified in a BD FACS Calibur flow cytometer. Three independent samples were analyzed for each condition.

For microscopy imaging, a volume of 25 µL of microencapsulated cells was rinsed twice in DPBS and re-suspended in 500 µL of 0.5 μM calcein AM and 0.5 μM ethidium homodimer-1 in DPBS. Next, solutions were placed in a 96-well plate and incubated at room temperature protected from light for 45 min. Samples were observed under a Nikon TMS confocal microscope at the wavelength of excitation 495 nm/emission 515 nm (for calcein AM staining) and excitation 495 nm/emission 635 nm (for ethidium homodimer staining). Random images were analyzed with the Eclipse Net software, version 1.20.0.

### Metabolic activity assay

Encapsulated cell metabolic activity was quantified at day 1 and 7 post-encapsulation. Five microliters per well of each encapsulated cell type were placed in 100 µl of medium on 96-well plates. Encapsulated cells in alginate without GO were used as controls. At least six wells were placed for each condition. Afterwards, 10 µl of Cell Counting Kit-8 CCK-8 solution (Sigma Aldrich) were added to each well. Plates were incubated inside a humidified chamber for 4 h at 37 °C and read on an Infinite M200 TECAN microplate reader at 450 nm with reference wavelength at 650 nm. At least three independent experiments were analyzed for each GO concentration.

### Toxicology assay based on lactic dehydrogenase (LDH) detection

The membrane integrity of encapsulated cells was quantified with the in vitro toxicology assay kit, LDH based (Sigma-Aldrich) 1 and 7 d after encapsulation. A total of 500 µl of medium was incubated with 50 µl of each type of encapsulated cells for 24 h, and 50 µl of supernatants were collected to determine LDH release activity. At the same time, 50 µl of each encapsulated cell type were incubated for 24 h with 500 µl of medium and lysed to determine the total LDH activity. All collected media were subjected to reaction with tetrazolium dye following manufacturer's recommendations. The resulting colored compound absorbance was read out on an Infinite M200 TECAN microplate reader at a wavelength of 490 nm, reading at 690 nm absorbance as background. At least three independent experiments were analyzed for each GO concentration.

### EPO and VEGF quantification

The secretion of EPO and VEGF for 24 h was quantified from each encapsulated D1-MSCs-EPO cell condition, 1 and 7 d after cell encapsulation. From encapsulated C_2_C_12_-EPO cells, only EPO secretion was quantified following the same procedure. A volume of 100 µL of microcapsules were rinsed twice with culture medium, re-suspended in 1 mL of medium and incubated for 24 h at 37 °C and 5% CO_2_. Then, supernatants were collected. Next microcapsules were cultured for 7 d, changing medium every 2 d, and rinsing twice 24 h before collecting supernatant at the end of the culture. The EPO secretion from the supernatants was quantified by Quantikine IVD EPO ELISA kit (R&D Systems, Minneapolis, MN, USA) while the secretion of VEGF was quantified by Human VEGF Standard ABTS ELISA Development Kit (Peprotech, London, UK) following manufacturer recommendations. Three independent samples and controls for each condition were assayed.

### Implantation and retrieval of encapsulated cells

Six weeks old allogenic Balb/c and C3H mice were purchased from Janvier Labs (Le Genest-Saint-Isle, France) and housed with sterile feed and autoclaved water. The following four different groups of alginate microcapsules containing C_2_C_12_-EPO cells or D1-MSCs-EPO cells were produced and implanted into 6 weeks old Balb/c or C3H mice, respectively: 380 µm diameter alginate microcapsules, 380 µm diameter alginate microcapsules containing 50 µg/ml of protein coated-GO, 160 µm diameter alginate microcapsules and 160 µm diameter alginate microcapsules containing 50 µg/ml of protein coated-GO. All the microcapsules were manufactured 1 d before implantation. Five animals per group were implanted subcutaneously with 200 µl of the respective encapsulated cells suspended in Hank´s Balanced Salt Solution in a final volume of 1 ml using a 18-gauge catheter. During the procedure, all animals were maintained under anesthesia by isofluorane inhalation. Blood samples were collected weekly by facial vein puncture with heparinized capillary tubes (Deltalab, Barcelona, Spain), and capillaries were spun down at 760 × *g* for 15 min. Hematocrits were determined using a standard microhematocrit method and expressed as mean ± standard deviation. Animals were sacrificed 6 weeks after implantation and the foreign body capsules containing microcapsules were retrieved. All the experimental procedures were performed in compliance with protocols approved by the institutional animal care and use committee of the University of Basque Country UPV/EHU (Permit Number: CEEA/360/2014/CIRIZA ASTRAIN).

The retrieved fibrotic capsules containing microcapsules from at least 3 implanted mice with each type of encapsulated cells were disaggregated by incubation at 37 °C for 3–4 h with sterile 2 mg/ml collagenase H and 1 mg/ml hyaluronidase dissolved in DMEM. The disaggregated suspension was filtered through a 70-µm strainers into a 50 ml conical tube, collecting microencapsulated cells on the top of the strainers and the solution containing the infiltrated cells from the fibrotic capsules in the conical tube. Next, infiltrated cells were collected by spinning down the disaggregated filtered solution, storing supernatant for further analysis, and re-suspending the pellet with medium into a single cell suspension for flow cytometry analysis.

### Metabolic activity and cell viability of retrieved encapsulated cells

Metabolic activity was quantified from 25 µl of each retrieved microencapsulated cell sample with the Cell Counting Kit-8 CCK-8 (Sigma Aldrich), following the same procedure described above. For cell viability, 25 µl of each retrieved microencapsulated cell type were rinsed twice in DPBS and stained with calcein AM and ethidium homodimer-1 as previously described.

### FACS analysis

For flow cytometry analysis, freshly single cell suspensions from solutions containing the infiltrated cells from the fibrotic capsules were stained with anti-CD16/32 antibody to block FcγII/III receptors. Fc blockage was followed by a 15-min staining with either an antibody cocktail containing PE-Cy7-conjugated anti-CD45, FITC-conjugated anti-CD3, PE-conjugated anti-CD4 and APC-conjugated anti-CD8 or the cocktail containing APC-conjugated anti-CD11b, PE-conjugated anti-CD19, APC-Cy7-conjugated anti-NK1.1and PE-Cy7-conjugated anti-CD45. After rinsing, DAPI (6 µM final concentration) was added before analysis to exclude dead cells. At least 10^6^ cells/sample were analyzed using a MACSQuant Analyzer 10 flow cytometer controlled by MACSQuantify™ Software’s (Miltenyi Biotec S.L., Bergisch Gladbach, Germany). Unstained cells were used to evaluate autofluorescence while fluorescent minus one controls were used to determine gates. Each experiment was carefully compensated by single staining single suspensions with the appropriate antibody: fluorochrome combination. In all cytometry experiments, at least three samples were analyzed for each condition.

### MagPix

Cytokines were assayed using a Luminex Magpix-based assay (Luminex Corporation, Austin, TX). Twenty-three cytokines (Eotaxin, G-CSF, GM-CSF, IFN-γ, IL-1α, IL-1β, IL-2, IL-3, IL-4, IL-5, IL-6, IL-9, IL-10, IL-12 (p40), IL-12 (p70), IL-13, IL-17A, KC, MCP-1 (MCAF), MIP-1α, MIP-1β, RANTES, and TNF-α) from the Bio-Plex ProT Mouse Cytokine 23-plex Assay (Bio-Rad, Hercules, CA, USA) were analyzed. The supernatant collected from at least three disaggregated retrieved fibrotic capsules containing microcapsules were thawed at 4 °C, centrifuged at 1400 × *g* to remove any aggregate protein that may potentially obstruct the measurement and transferred to a fresh tube. An 8-point standard curve with serial dilutions of 1:4 was generated using reconstituted stock standards supplied by the manufacturer; quality controls supplied by the manufacturer were also used to determine assay accuracy. The data were generated using the Bio-Plex Manager Software (Bio-Rad, Hercules, CA, USA). All samples were assayed in duplicate. The average coefficient of variance for duplicate values across analytes for samples was 5%. The average minimum detectable value across all plates was 0.56 pg/ml for IL-1a, 2.07 pg/ml for IL-1b, 0.98 pg/ml for IL-2, 0.46 pg/ml for IL-3, 0.4 pg/ml forIL-4, 0.79 pg/ml for IL-5, 0.7 pg/ml for IL-6, 2.4 pg/ml for IL-9, 3.77 pg/ml for IL-10, 8.33 pg/ml forIL-12(p40), 20.22 pg/ml for IL-12(p70), 11.11 pg/ml for IL-13, 0.41 pg/ml for IL-17A, 7.9 pg/ml for Eotaxin, 3.78 pg/ml for G-CSF, 4.44 pg/ml for GM-CSF, 0.8 pg/ml for IFN-γ, 1.22 pg/ml for KC, 45.33 pg/ml for MCP-1,0.44 pg/ml for MIP-1a, 3.14 pg/ml for MIP-1b, 1.91 pg/ml for RANTES and 4.44 pg/ml for TNF-α.

### Immunohistochemistry

The retrieved fibrotic capsules containing microcapsules were included in OCT (VWR chemicals, Radnor, PA, USA), frozen and kept at −80 °C until their study. About 10 µm cryosections were cut and stained with hematoxylin and eosin and Masson’s trichrome. Microscopy sections were examined by a pathologist/expert blinded to the treatments. The presence and distribution of infiltrating cells, preservation, and vascularization of the tissue along with the extension of fibrosis were evaluated.

### Statistical analysis

Statistical analysis was performed using SPSS software, version 21.00.1, or GraphPad Prism 5.01 (GraphPad Inc., San Diego, CA, USA). Data are expressed as a mean ± standard deviation. Values from *p* < .05 to *p* < .001 were considered significant for comparison of groups using ANOVA, Tukey’s *post hoc* test or Kruskal–Wallis H test.

## Results and discussion

### Doubling diameter of hybrid alginate-protein-coated GO microcapsules enhances encapsulated myoblasts viability and protein release

First, we aimed to study the behavior of encapsulated genetically modified C_2_C_12_ myoblasts within hybrid alginate-protein-coated GO microcapsules (hybrid microcapsules) with diameters higher than 300 µm, trying to determine if an increment in size would affect the benefits observed with 160 µm diameter hybrid microcapsules (Saenz Del Burgo et al., 2017). We compared viability, metabolic activity, cell membrane integrity and protein release of encapsulated C_2_C_12_-EPO cells in 160 and 380 µm diameter hybrid microcapsules, performed in a pneumatic (Bioencapsulation portable platform Cellena^®^) and an electrostatic (Nisco^®^) atomization generator, respectively (Supplementary Figure 1). We generated both diameter hybrid microcapsules at a concentration of 50 µg/ml of GO, comparing them with microcapsules without GO, since this GO concentration within alginate microcapsules has previously shown to be the optimal concentration for myoblasts survival enhancement (Ciriza et al., 2015). All hybrid microcapsules presented a homogenous spherical shape.

**Figure 1.**
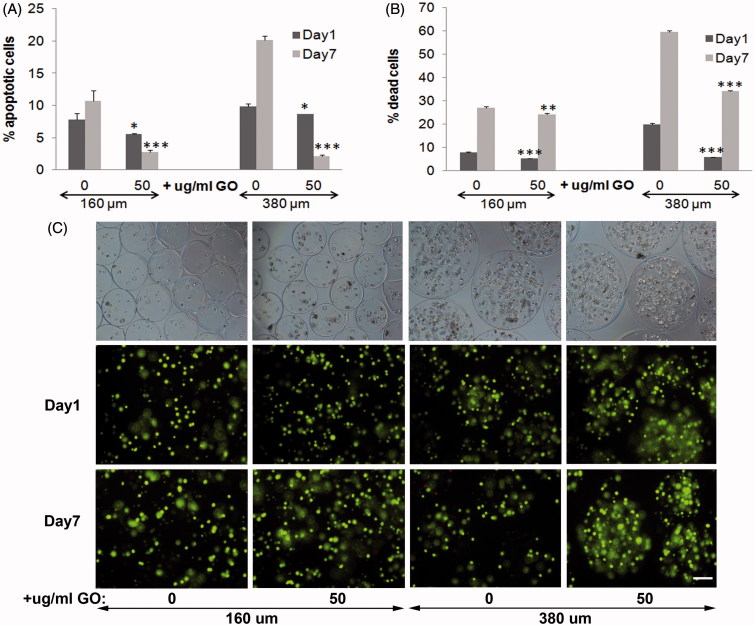
Viability of encapsulated C_2_C_12_-EPO myoblasts within 160 and 380 µm diameter alginate (0 µg/ml) and hybrid alginate-GO microcapsules (50 µg/ml), 1 and 7 d after microencapsulation: (A) Fold reduction of apoptotic cell percentage compared to microcapsules without GO quantified by flow cytometry of early apoptosis by means of annexin/PI staining and (B) Fold reduction of dead cell percentage compared to microcapsules without GO quantified by flow cytometry after calcein/ethidium staining. (C) Fluorescence and bright field microscopy images after calcein/ethidium staining. Scale bar 100 µm. Note *p < .05, **p < .01, and ***p < .001 compared with cells encapsulated in alginate without GO.

Next day and 7 d after encapsulation, we quantified significantly lower apoptotic cell population (*p* < .05 and *p* < .001, respectively) in presence of GO with both diameters of microcapsules, compared to their counterparts without GO. Although we confirmed apoptosis reduction both days after encapsulation caused by the presence of protein-coated GO within 160 µm diameter microcapsules (Saenz Del Burgo et al., 2017), significant higher apoptotic cell percentage reduction (*p* < .001) was quantified with 380 µm diameter hybrid microcapsules 7 d after encapsulation (Figure 1(A)). These results indicated that the presence of protein-coated GO in 380 µm diameter microcapsules improves the beneficial effect detected in smaller microcapsules. Viability quantification assays reinforced apoptosis results (Figure 1(B)). Both diameters of GO coated encapsulated C_2_C_12_-EPO cells showed significantly lower dead cell percentage (*p* < .001) the next day and 7 d after encapsulation, compared with their counterparts without GO. However, the reduction in dead cell percentage was significantly higher in 380 µm diameter microcapsules than in 160 µm (*p* < .001), confirming again the beneficial improvement of the combination of protein-coated GO and bigger diameter microcapsules. These results were verified under fluorescent microscope after staining encapsulated cells with calcein/ethidium (Figure 1(C)), confirming that the presence of protein-coated GO reduces apoptosis and cell death in alginate microcapsules with a more accused effect in 380 µm diameter microcapsules.

**Figure 2.**
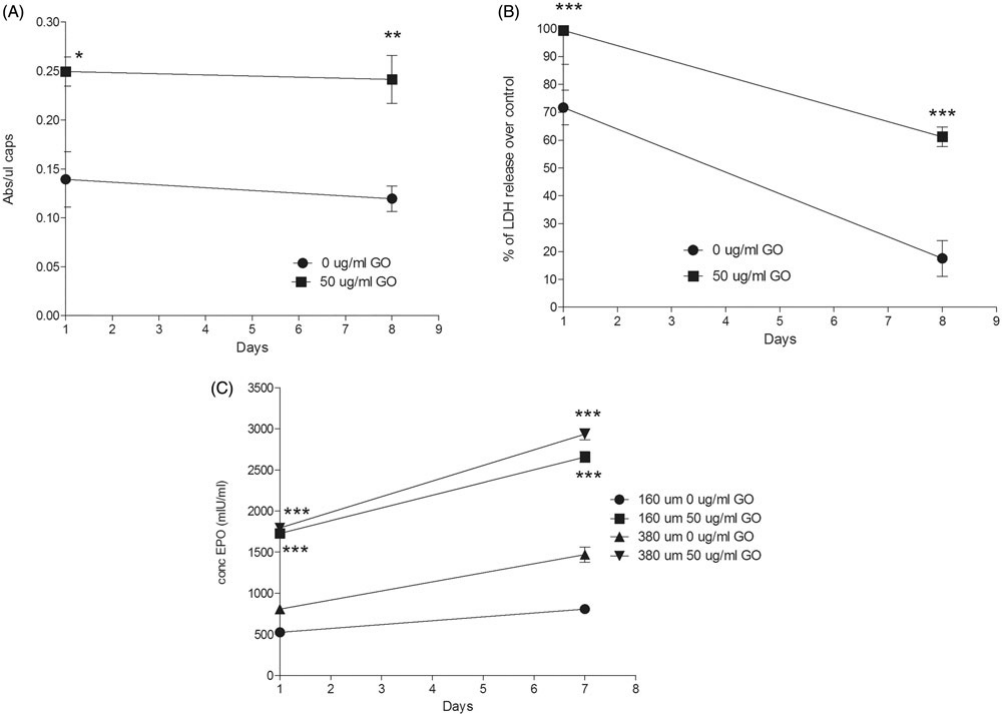
(A) Metabolic activity and (B) cell membrane integrity of encapsulated C_2_C_12_-EPO myoblasts within 380 µm diameter alginate and hybrid alginate-GO microcapsules (50 µg/ml), 1 and 7 d after microencapsulation. (C) EPO secretion 1 and 7 d after microencapsulation of C_2_C_12_-EPO myoblasts within 160 and 380 µm diameter alginate and hybrid alginate-GO microcapsules (50 µg/ml). Note *p < .05, **p < .01, and ***p < .001 compared with cells encapsulated in alginate without GO.

Metabolic activity quantification also confirmed the beneficial effect of the presence of GO nanoparticles. Similarly to 160 µm diameter hybrid microcapsules (Saenz Del Burgo et al., 2017), metabolic activity of encapsulated C_2_C_12_-EPO myoblasts within 380 µm diameter hybrid microcapsules was significantly higher (*p* < .05) than their counterparts without GO (Figure 2(A)). However, the analysis of cell membrane integrity showed that the presence of protein-coated GO nanoparticles affected the encapsulated C_2_C_12_-EPO myoblasts membrane integrity (Figure 2(B)). When cell membrane is damaged, intracellular LDH is released into the medium, and therefore LDH levels reflect the integrity of the cell membrane. The significant detected increment (*p* < .001) in LDH leakage compared to microcapsules without GO, and therefore, the worse cell membrane integrity, suggest that protein-coated GO nanoparticles could provoke some toxicity to encapsulated C_2_C_12_-EPO myoblasts within 380 µm diameter-hybrid alginate- GO microcapsules, similar to the results previously described with graphene and single-wall carbon nanotubes (Zhang et al., 2010). However, like microcapsules without GO, LDH release decreased over time, with lower LDH release 7 d after encapsulation, indicating that any toxic effect of protein-coated GO nanoparticles within the microcapsules is overcome over time.

Finally, we quantified the release of the therapeutic agent EPO by encapsulated cells because the sustained drug delivery is one of the main advantages for preclinical applications offered by this encapsulation technology (Orive et al., 2005; Murua et al., 2009). We studied and compared the release of EPO produced by encapsulated C_2_C_12_-EPO myoblasts in the presence and absence of protein-coated GO nanoparticles. Both hybrid microcapsules showed a significant increment (*p* < .001) in EPO production and release compared to microcapsules without GO (Figure 2(C)). Since the presence of FBS-coated GO reduces the number of dead and apoptotic cells (Figure 1(A,B)), it can be concluded that the higher EPO release quantified is a consequence of an increment in the number of viable cells. Moreover, significant higher release was detected in C_2_C_12_-EPO myoblasts within 380 µm diameter hybrid microcapsules than in 160 µm at day 7 (*p* < .05), concluding that doubling hybrid alginate-protein-coated GO microcapsules size enhances encapsulated myoblasts sustained release *in vitro*.

### Viability of encapsulated MSCs within hybrid alginate-protein-coated GO microcapsules is also enhanced

**Figure 3.**
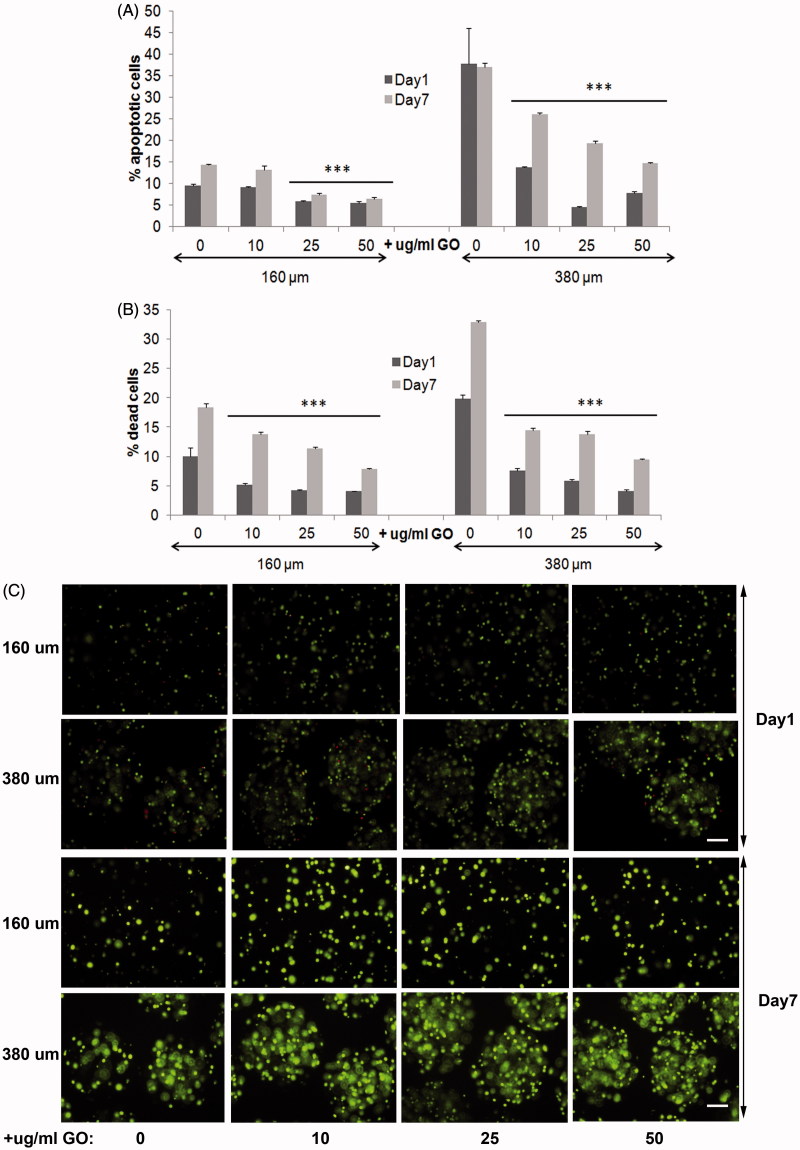
Viability of encapsulated D1-MSCs EPO within 160 and 380 µm diameter alginate and hybrid alginate-GO microcapsules (10, 25, and 50 µg/ml), 1 and 7 d after microencapsulation: (A) Quantification by flow cytometry of early apoptosis by means of annexin/PI staining and (B) live/dead percentage by means of calcein/ethidium staining. (C) Fluorescence microscopy images after calcein/ethidium staining. Scale bar 100 µm. Note ***p < .001 compared with cells encapsulated in alginate without GO.

Next, we studied the behavior of encapsulated MSCs within 380 µm and 160 µm diameter hybrid microcapsules before performing *in vivo* implantations, with the goal of further circumvent the *in vivo* immune rejection of hybrid alginate-GO encapsulated cells through the immune-modulation from MSCs of the microenvironment (Maccario et al., 2005; Meirelles Lda et al., 2009; Shi et al., 2011; Gebler et al., 2012). So, first, we studied the biocompatibility of different GO concentrations up to 50 µg/ml, which are required in alginate-GO cell microencapsulation for each cell type and therapeutic protein (Ciriza et al., 2015), since concentrations higher than 50 µg/ml difficult the manufacturing in the encapsulator devices. Thus, we produced encapsulated D1-MSCs-EPO cells in 160 and 380 µm diameter hybrid microcapsules with the pneumatic and the electrostatic atomization generator, respectively, at 10, 25, and 50 µg/ml of GO concentrations and 5 × 10^6^ cells/ml cell density to, next, compare their viability, metabolic activity, cell membrane integrity, and protein release with alginate microcapsules without GO. All hybrid microcapsules generated displayed spherical shape. We quantified lower apoptosis in 160 µm diameter microcapsules than in 380 µm, independently of the protein-coated GO presence or absence (Figure 3(A)). However, when comparing 380 µm diameter hybrid microcapsules with their counterpart without GO, the decrease in percentage of apoptotic cells was significantly higher (*p* < .001), than when comparing 160 µm diameter hybrid alginate- GO microcapsules with their counterpart without GO. These results indicate that the presence of protein-coated GO nanoparticles helps to reduce apoptotic processes. It is remarkable that similarly to encapsulated C_2_C_12_-EPO myoblasts (Saenz Del Burgo et al., 2017), and independently of the microcapsule diameter, protein-coated GO nanoparticles at 50 µg/ml was the concentration with the lowest apoptotic cell percentage. Viability quantification assays provided similar results than apoptosis. Both diameters of encapsulated D1-MSCs-EPO cells showed significantly lower death cell percentage (*p* < .001) than their counterparts without GO (Figure 3(B)), but the reduction in death cell percentage was also higher in 380 µm diameter microcapsules than in 160 µm, reinforcing the higher beneficial effect of the combination of protein-coated GO and 380 µm diameter microcapsules instead of 160 µm. Again, and similarly to encapsulated C_2_C_12_-EPO myoblasts (Saenz Del Burgo et al., 2017), protein-coated GO nanoparticles at 50 µg/ml showed the lowest dead cell percentage, independently of the microcapsule diameter. Micrographs displayed after staining encapsulated cells with calcein/ethidium confirmed these results (Figure 3(C)), and indicated that 50 µg/ml protein-coated GO reduces optimally apoptosis and cell death in alginate microcapsules, with an accused effect in 380 µm diameter microcapsules.

**Figure 4.**
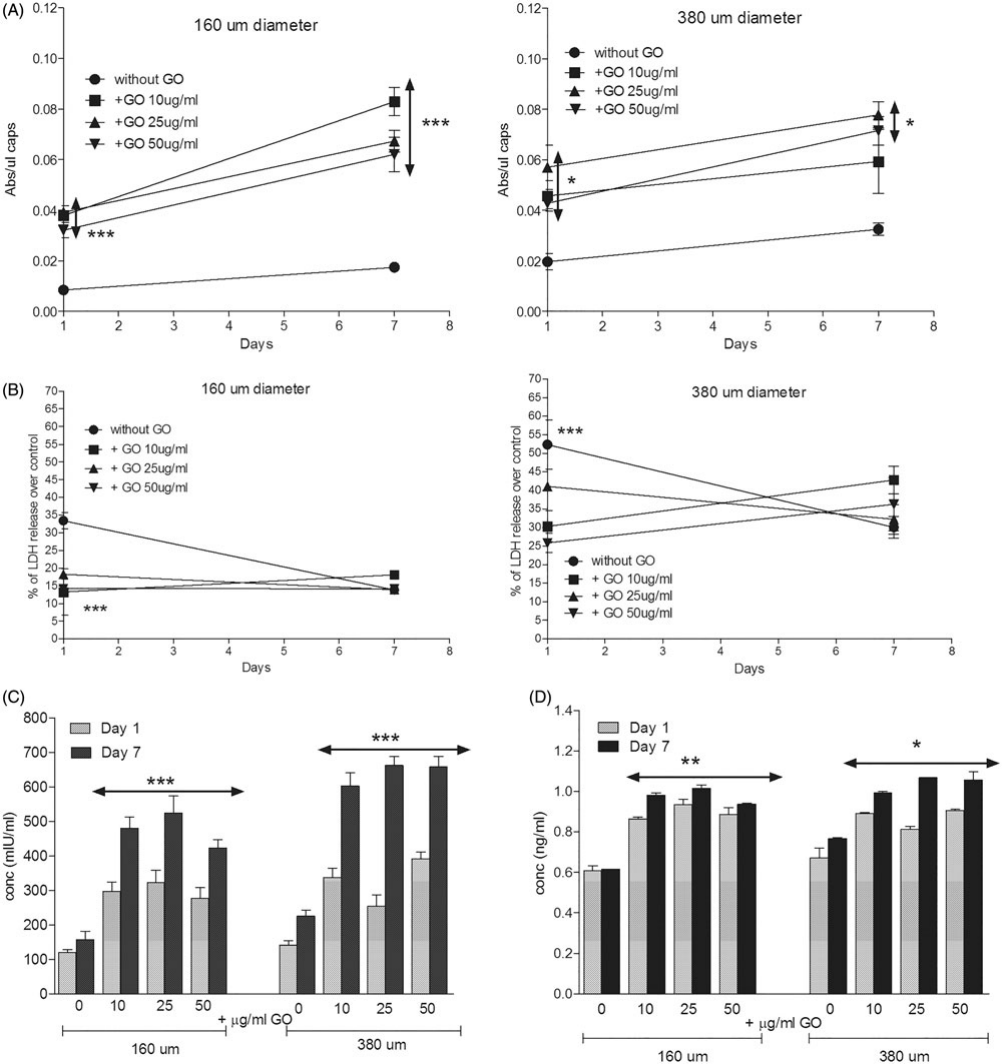
(A) Metabolic activity and (B) cell membrane integrity of encapsulated D1-MSCs EPO within 160 µm (left) and 380 µm (right) diameter alginate and hybrid alginate-GO microcapsules (10, 25, and 50 µg/ml), 1 and 7 d after microencapsulation. (C) EPO and (D) VEGF secretion 1 and 7 d after microencapsulation of D1-MSCs EPO within 160 and 380 µm diameter alginate and hybrid alginate-GO microcapsules (10, 25, and 50 µg/ml). Note *p < .05, **p < .01, and ***p < .001 compared with cells encapsulated in alginate without GO.

Metabolic activity was also significantly higher for all the encapsulated D1-MSCs-EPO cells in hybrid microcapsules compared to their counterparts without GO, independently of the diameter studied (Figure 4(A)) and similarly to encapsulated C_2_C_12_-EPO myoblasts (Ciriza et al., 2015; Saenz Del Burgo et al., 2017). However, different results were observed comparing the metabolic activity among the different protein-coated GO concentrations at both microcapsules diameters. In 160 µm diameter hybrid microcapsules, 10 µg/ml protein-coated GO provided no significant higher values of metabolic activity 7 d after encapsulation (Figure 4(A)). However, in 380 µm diameter hybrid microcapsules 25 µg/ml protein-coated GO provided the highest values of metabolic activity since the first day after encapsulation. In spite of these differences, all metabolic activities were higher than observed with microcapsules without GO (Figure 4(A)), confirming again the benefits of protein-coated GO nanoparticles. Similar results were shown when the membrane integrity was analyzed. Encapsulated D1-MSCs-EPO within both diameters of hybrid microcapsules displayed better membrane integrity than their counterparts without GO the first day after encapsulation, independently of the protein-coated GO concentration studied reaching similar membrane integrity values 7 d after encapsulation (Figure 4(B)). The beneficial effects observed from GO could be related to two factors. On one hand, the protective effect previously described by GO in 3 D matrixes (Ciriza et al., 2015). On the other hand, since GO is able to adsorb the 25% of the serum proteins, such as some extracellular matrix globular proteins and glycoproteins like fibronectin (Cedervall et al., 2007; Dell'Orco et al., 2010; Lee et al., 2011; Saenz Del Burgo et al., 2017), FBS coating could enhance GO protective effect by facilitating cell adhesion, similarly to the RGD (motif from fibronectin) modified alginates (Orive et al., 2009, Garate et al., 2015).

D1-MSCs-EPO cells are genetically engineered to secrete EPO (Gurruchaga et al., 2015). Thus, we quantified the sustained drug delivery of this therapeutic agent in the hybrid microcapsules. Cells encapsulated within hybrid microcapsules at both diameters released significantly higher (*p* < .001) amount of the therapeutic protein than alginate microcapsules without GO 7 d after encapsulation (Figure 4(C)), similarly to encapsulated C_2_C_12_-EPO myoblasts (Ciriza et al., 2015; Saenz Del Burgo et al., 2017). Moreover, doubling the hybrid microcapsules size also enhanced the release of the therapeutic protein synthesized by the encapsulated MSCs *in vitro*. Interestingly, 380 µm diameter hybrid alginate-GO microcapsules containing 50 µg/ml protein-coated GO released no significant higher amount of EPO overtime, while 160 µm diameter hybrid microcapsules containing 25 µg/ml protein-coated GO released more amount of EPO overtime (Figure 4(C)). However, no significant differences were detected among the different GO concentrations in both diameter hybrid microcapsules. Because the presence of protein-coated GO nanoparticles in alginate microcapsules could also be influencing the release of endogenous proteins secreted by MSCs, we also compared the endogenous release of VEGF (Beckermann et al., 2008). Hybrid microcapsules released significantly more VEGF (*p* < .05) than alginate microcapsules without GO, independently of the GO nanoparticles concentration within both microcapsule diameters (Figure 4(D)). This increment of VEGF release was correlated with the increment of EPO secretion, indicating that the secretion enhancement could be influenced by the boost of metabolic activity through GO, similarly to alginate microcapsules containing hyaluronic acid (Canibano-Hernandez et al., 2017).

### Doubling the diameter of hybrid alginate-protein-coated GO microcapsules enhances EPO release by encapsulated cells *in vivo*

**Figure 5.**
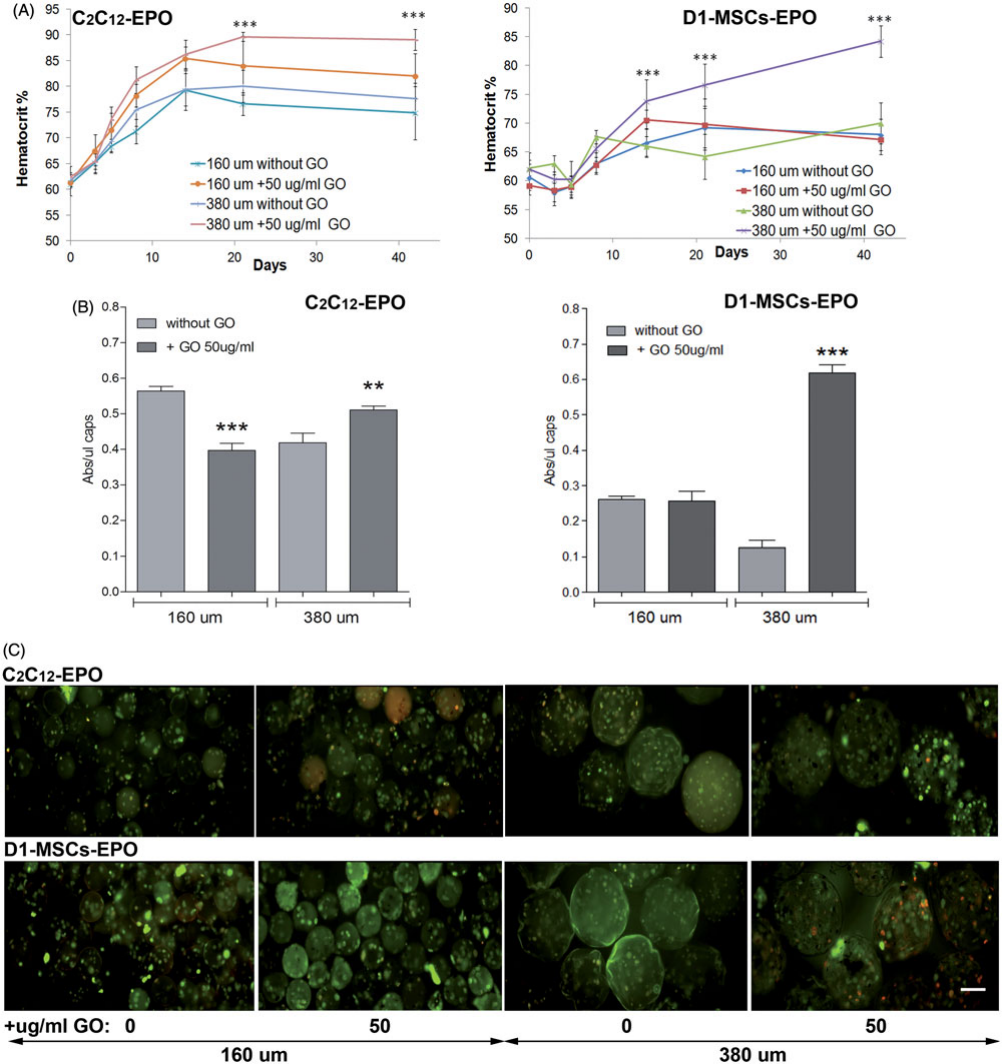
Subcutaneous allogenic implantation of encapsulated C2C12-EPO myoblasts and D1-MSCs EPO within 160 and 380 µm diameter alginate and hybrid alginate-GO microcapsules (50 µg/ml). (A) Hematocrit levels in C3H (left) and Balb/c (right) mice expressed as mean + SD after allogenic implantation. (B) Metabolic activity of retrieved encapsulated C2C12-EPO myoblasts from C3H mice (left) and encapsulated D1-MSCs EPO from Balb/c mice (right). (C) Fluorescence microscopy images after calcein/ethidium staining of the retrieved encapsulated cells. Scale bar 200 µm. Note **p < .01 and ***p < .001 compared with cells encapsulated in alginate without GO.

After observing the increment of EPO release in either encapsulated C_2_C_12_-EPO myoblasts or D1-MSCs-EPO within 160 or 380 µm diameter hybrid microcapsules, we wonder if the EPO release increment quantified in both encapsulated cell types and hybrid microcapsules diameters would be reflected in murine hematocrit levels after microcapsules implantation. We implanted, therefore, 200 µl of encapsulated C_2_C_12_-EPO myoblasts or D1-MSCs-EPO within 160 and 380 µm diameter hybrid (50 µg/ml) microcapsules into allogeneic Balb/c and C3H mice, respectively, and determined their blood hematocrit levels for 6 weeks, comparing with mice implanted with the respective microcapsule without GO. Three weeks after implantation, the hematocrit significantly (*p* < .001) increased in allogeneic mice implanted with encapsulated cells within 380 µm diameter hybrid alginate- GO microcapsules (Figure 5(A)). Mice implanted with both encapsulated cell types within 160 µm diameter hybrid microcapsules did not significantly increase hematocrit blood levels compared to their counterparts without GO, in contrast to expected from EPO release *in vitro* results or the significant hematocrit blood level increment described after implantation of encapsulated C_2_C_12_-EPO myoblasts within 160 diameter hybrid microcapsules into syngeneic mice (Saenz Del Burgo et al., 2017). We also quantified the metabolic activity of the retrieved microcapsules 6 weeks after implantation. Similarly to *in vitro* studies with encapsulated C_2_C_12_-EPO myoblasts (Figure 2(A)) and D1-MSCs-EPO (Figure 4(A)) within 380 µm diameter hybrid microcapsules, retrieved hybrid microcapsules showed significantly higher metabolic activity (*p* < .005) than their counterparts without GO (Figure 5(B)). However, retrieved encapsulated cells within 160 µm diameter hybrid microcapsules displayed reduced metabolic activity than their counterparts without GO, with more significant pronounced effect (*p* < .001) in hybrid microcapsules containing C_2_C_12_-EPO myoblasts than those containing D1-MSCsEPO (Figure 5(B)). These results of metabolic activity were corroborated by microscopy images obtained after calcein/ethidium staining (Figure 5(C)), indicating that 160 µm diameter hybrid microcapsules do not behave similarly to *in vitro* conditions. This lower metabolic activity of 160 µm diameter hybrid microcapsules would explain the lack of significant increment in the hematocrit of implanted allogeneic mice when compared to mice implanted with the controls without GO. In fact, one of the major challenges in the use of allogeneic cells is their potential transplant rejection (Li & Duncan, 1998; Tambuyzer et al., 2009). The hematocrit of syngeneic recipients implanted with no encapsulated MSCs engineered to release murine EPO rapidly rises from baseline level, remaining high for more than 200 d, while in MHC-mismatched recipient mice, the hematocrit transiently rises, rapidly declining to baseline values (Eliopoulos et al., 2005). Allogeneic non encapsulated MSC implants shows increased infiltration of CD8 and NK cells compared to syngeneic controls, with a significant interferon-gamma response in the spleens (Eliopoulos et al., 2005). So, we hypothesized that although 500 µm diameter alginate microcapsules loaded with C_2_C_12_-EPO myoblasts and subcutaneously implanted in allogeneic mice are able to maintain high and constant hematocrit levels for more than 100 d (Orive et al., 2005), immune system could affect more to smaller microcapsules, with diameters of 160 µm, creating a similar scenario to the aforementioned allogeneic implantation of MSCs secreting EPO (Eliopoulos et al., 2005).

### Modifying the diameter of hybrid alginate-protein-coated GO microcapsules does not affect foreign body reaction

**Figure 6.**
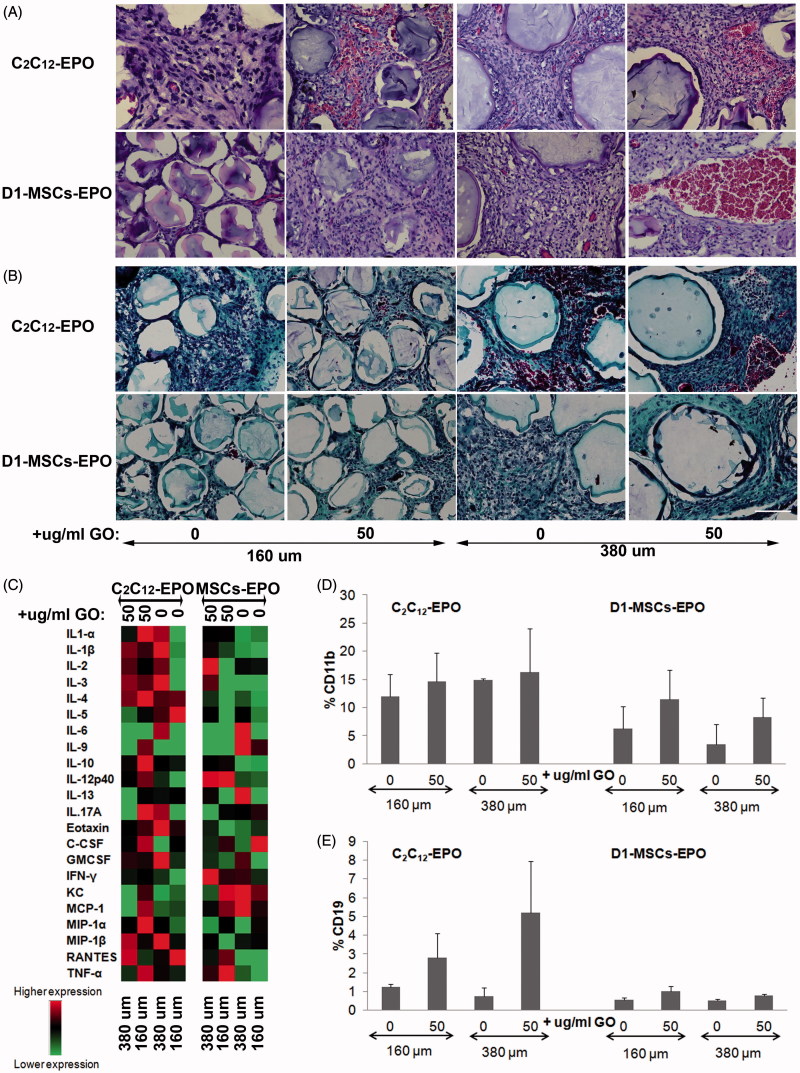
Foreign body reaction analysis 6 weeks after subcutaneous allogenic implantation of encapsulated C2C12-EPO myoblasts and D1-MSCs EPO within 160 and 380 µm diameter alginate and hybrid alginate-GO microcapsules (50 µg/ml). (A) Representative photographic images of hematoxylin-eosin staining of explanted grafts after 6 weeks (B) Representative photographic images of Masson's trichrome staining of explanted grafts after 6 weeks (C) Expression analysis of 23 inflammation-related cytokines by means of Bio-Plex Pro Mouse Cytokine 23-Plex Immunoassay. Flow cytometry quantification of D) CD11b and (E) CD19 percentage infiltrated cells from the retrieved fibrotic capsules. Note: Scale bar 100 µm.

Recent studies have shown that tuning spherical dimensions improves the biocompatibility of biomedical devices, demonstrating that a three-fold increment of alginate capsules prolongs the period of blood glucose restoration in streptozotocin-treated diabetic C57BL/6 mice five times longer by mitigating foreign reactions and fibrosis (Veiseh et al., 2015). We studied, therefore, if the microcapsule size, the presence of protein-coated GO (50 µg/ml) within the microcapsules or the cell type within the microcapsules could induce a stronger immune response after allogeneic implantation. We easily recovered in one piece the explanted grafts from MHC-mismatched mice 6 weeks after implantation of either encapsulated C_2_C_12_-EPO myoblasts or D1-MSCs-EPO within 160 or 380 µm diameter hybrid microcapsules. The microcapsule network from all the groups was surrounded by blood capillaries showing a weak fibrotic layer surrounding the APA microcapsules in the histological analyses. Histological evaluation of the explanted microcapsules evidenced the formation of pericapsular overgrowth maybe due to the direct contact of the pro-inflammatory PLL at the surface of APA microcapsules with cells of the host immune response (Tam et al., 2005, 2011). Hematoxylin-eosin staining evidenced the presence of erythrocytes in all the grafts with higher preponderance in samples from allogeneic animals implanted with microcapsules containing GO (Figure 6(A)), with the exception of 160 µm diameter hybrid microcapsules containing D1-MSCs-EPO and in accordance with the hematocrit increment observed (Figure 5(A)). Moreover, samples with higher presence of erythrocytes were accompanied by higher vascularization, confirming that the presence of GO in hybrid microcapsules improves the release of EPO *in vivo*. Masson’s trichrome staining showed fibrotic tissue in all the samples implanted without differences visualized among the different grafts, independently of the microcapsules implanted (Figure 5(B)). Fibrin staining could be detected in grafts from implantations with encapsulated C_2_C_12_-EPO myoblasts, indicating that D1-MSCs-EPO could slightly attenuate the initiation of the foreign body reaction compared to encapsulated C_2_C_12_-EPO myoblasts. However, the data indicate that the inclusion of FBS coated GO does not provoke a significant modification in the immune reaction compared to the immune reaction induced by alginate microcapsules without GO.

Next, we quantified the protein expression of 23 inflammation-related cytokines within the supernatant collected from at all the retrieved grafts containing microcapsules (Figure 6(C)). No significant differences were detected in the expression of all the cytokines among all the explanted grafts. However, higher expression of pro-inflammatory cytokines was detected in grafts from encapsulated C_2_C_12_-EPO myoblasts compared to encapsulated D1-MSCs-EPO implanted allogeneic mice, especially when comparing GO containing counterparts. These results would suggest slight immune response attenuation in the surrounding environment by encapsulated D1-MSCs-EPO in terms of pro-inflammatory cytokines secretion.

We also quantified in all the retrieved grafts the percentage of the following infiltrated cell populations by flow cytometry: CD45^+^CD3^+^CD4^+^CD8^-^, CD45^+^CD3^+^CD4^-^CD8^+^, CD11b^+^, CD19^+^, and NK1.1^+^. No significant differences in the percentage of any of the infiltrated cell population studied were detected among the samples retrieved from mice (data not shown), indicating that the studied immune cell populations do not significantly affect microcapsules with different size or with GO. Although the host response kinetic profile 1 month after subcutaneous or intraperitoneal implantation have previously shown a reduced significant percentage of macrophages and neutrophils in 1.5 mm implanted spherical capsules compared to 0.5 mm capsules (Veiseh et al., 2015), we did not detect significant immune cell population differences after implantation by tuning our microcapsules size to higher diameters. This lack of differences could reside in the different capsule sizes studied, since our microcapsules range 160–380 µm, instead of 0.5–1.5 mm, which could give rise to different host response kinetic profile. Moreover, the studied host strains, Balb/c and C3H, could also provoke different host response kinetic profile compared to the previously described implanted C57BL/6 mouse strain, which produces stronger fibrotic and foreign body response (Kolb et al., 2002). Another factor that could modify the host kinetic profile is the presence of contaminating endotoxins, which induce stronger foreign body reaction (Paredes-Juarez et al., 2013). Finally, the protein release profile from different cell types could also influence the immune response, with different host response against encapsulated rat islets (Veiseh et al., 2015) than encapsulated myoblasts or MSCs. In fact, MSCs are able to immune-modulate the microenvironment by inhibiting the function of different immune cell subpopulations of the innate and adaptive immunity (Shi et al., 2011), e.g. suppressing T cell proliferation through the secretion of PGE-2, IDO, and TGF-β (Meirelles Lda et al., 2009), inducing CD4 + CD25 + FoxP3 + and CD8 + regulatory cells (Maccario et al., 2005), blocking B cells proliferation in presence of IFN-γ (Gebler et al., 2012) and down-regulating the co-stimulatory molecules CD40, CD80, and CD86 expression on dendritic cells (Nauta et al., 2006). Interestingly, we detected lower CD11b^+^ and CD19^+^ infiltrated cell percentage in mice implanted with encapsulated D1-MSCs-EPO than with C_2_C_12_-EPO myoblasts. These not significant differences were independent of the capsule diameter or the presence/absence of protein coated-GO nanoparticles (Figure 6(D–E)) suggesting that MSCs can modulate the essential innate (macrophage) and adaptative (B cell) immune population in the fibrotic response to implanted alginate microcapsules (Doloff et al., 2017). Stronger fibrotic and foreign body response from different murine strains, such as C57BL/6 (Kolb et al., 2002) could have provided a more accused significant percentage reduction of CD11b^+^ and CD19^+^ infiltrated cell after allogeneic implantation. We believe that the combination of new hydrogel surfaces (Vegas et al., 2016) and encapsulated MSCs could mitigate the foreign body response without the need of immune suppression regimes, or the specific blockade or inhibition of chemokines and/or cytokines involved in the recognition and propagation of foreign body rejection response to hydrogels (Doloff et al., 2017).

## Conclusions

We have corroborated that the presence of 50 µg/ml protein-coated GO nanoparticles within alginate microcapsules reduces apoptosis and increase cell survival, metabolic activity, and therapeutic protein release of encapsulated myoblasts and MSCs. Moreover, we have demonstrated that a two-fold size increment of alginate microcapsules diameter enhances even more the beneficial effects created by the presence of GO within microcapsules. Importantly, this significant improvement of therapeutic protein release in hybrid alginate-protein-coated GO microcapsules is directly translated *in vivo*, providing a quicker and higher physiological outcome.

Interestingly, the encapsulation of MSCs suggests a reduction of innate and adaptative immune population in the foreign body response against hydrogels, without the need of immune suppression, while the presence of protein-coated GO nanoparticles does not induce a higher immune response. We can conclude that protein-coated GO nanoparticles provide a suitable microenvironment for encapsulated cells and represent a good alternative to improve the sustained drug release from microcapsules, with better outcomes in the more extensively evaluated 380 µm diameter microcapsules.
